# Utilization of dental care among adult populations: a scoping review of applied models

**DOI:** 10.1186/s12903-023-03323-1

**Published:** 2023-08-27

**Authors:** Ashkan Negintaji Zardak, Mostafa Amini-Rarani, Ibrahim Abdollahpour, Faezeh Eslamipour, Bahareh Tahani

**Affiliations:** 1 Oral Public Health Department, Dental school, Isfahan University of Medical Sciences, Isfahan, Iran; 2 Social Determinants of Health Research Center, Isfahan University of Medical Sciences, Isfahan, Iran; 3 Child Growth and Development Research Center, Research Institute for Primordial Prevention of Non-communicable Disease, Isfahan University of Medical Sciences, Isfahan, Iran; 4 Department of Oral Public Health, Dental Research Center, Dental Research Institute, Dental School, Isfahan University of Medical Sciences, Isfahan, Iran

**Keywords:** Health care utilization, Dental care, Oral health, Dental health services

## Abstract

**Background:**

The timely and appropriate utilization of dental health care is essential to the prevention and accurate treatment of oral diseases. Therefore, it is crucial that managers, health professionals and healthcare providers be fully aware of the predictors encouraging the utilization of dental services and reduce social inequalities. In this scoping review, we aimed to analyze the published articles and reports to find out the factors associated with dental services utilization and the comprehensiveness of the applied models among general adult populations.

**Materials and methods:**

This scoping study was based on the 5-steps of Arksey and O’Malley framework. Keywords were selected under two main concepts: determinants of dental care utilization and the concept of the applied models. Searches were conducted in some electronic databses including PubMed, Google Scholar and Scopus with variations, and a combination of the keywords under the two main afore-mentioned concepts. All the relevant articles reporting the utilization of dental care and its potential predictors among adult populations were chosen. No restrictions involving terms of study time, location or methodological aspects of oral health utilization were considered. Using tables and charts mapping, we tried to group the studies based on the year of their publication, geographic distribution, the range of included indices and the type of their measurement. Also, a directed content analysis method was used to investigate the comprehensiveness of the studies in regard to considering the determinant factors at different levels suggested by the Andesen model.

**Results:**

Fifty-two articles were included in the analysis. Thirty-six (69%) had been published between 2016 and 2020. The United States had conducted the most research in this scope. About 30% of studies had mentioned all three domains of demographics, social structure and beliefs, simultaneously. To evaluate the enabling factors, in 84.61% and 59.61% of studies, the income levels and insurance feature were assessed, respectively. 57.69% of the retrieved studies considered the perceived need features and 38.46% referred to the evaluated ones. The dental services utilization, in terms of the last visit during the “past 12 months”, was assessed more commonly. Only 11.54% of studies did evaluate the contextual characteristics and about 71.15% of articles were relatively comprehensive.

**Conclusion:**

Overall, it seems that in most of the studies, not all of the determinant factors at different levels of the Andersen model have been considered. In order to discover the conceptual linkages and feedback loops of the model, it is essential to conduct more comprehensive research in the future.

**Supplementary Information:**

The online version contains supplementary material available at 10.1186/s12903-023-03323-1.

## Background

Oral diseases are global public health concerns due to their prevalence and impact on individuals and societies [1]. According to the Global Burden of Disease report, around 3.5 billion people live with oral diseases worldwide [2]. Oral health status is associated with physical and cognitive functions, and numerous chronic diseases including diabetes and cardiovascular diseases [3]. Disadvantaged and socially marginalized populations, particularly in low- and middle-income countries, are mostly affected by the burden of oral conditions [4, 5].

The timely and appropriate utilization of dental health care is essential to the prevention and treatment of oral diseases; it is, therefore, necessary to identify the factors facilitating or impeding the dental care utilization. These determinants have been the subject of many research studies worldwide [6–9]. The utilization of health services results from the interaction between individual and contextual factors, including access to health services and organization of the health care system [7]. It has been shown that individuals living in socially deprived communities, such as rural areas, have less access to health services and experience poorer health status than those from more affluent communities [10].

Based on the results of the National Survey in the USA (NHANSE), low income, poor health, and uninsured women were more likely to report unmet dental care needs, suggesting the expansion of insurance coverage for dental care and improvement of the access for women with poor health to address racial-ethnic and education-level disparities in regard to unmet dental care needs [11]. Center for Oral Health Research in Appalachia also suggests state residency, sex, insurance, income, fatalistic beliefs, health values, and aspects of dental care-related anxiety and fear as the factors predicting dental care utilization [12].

**Fig. 1:**
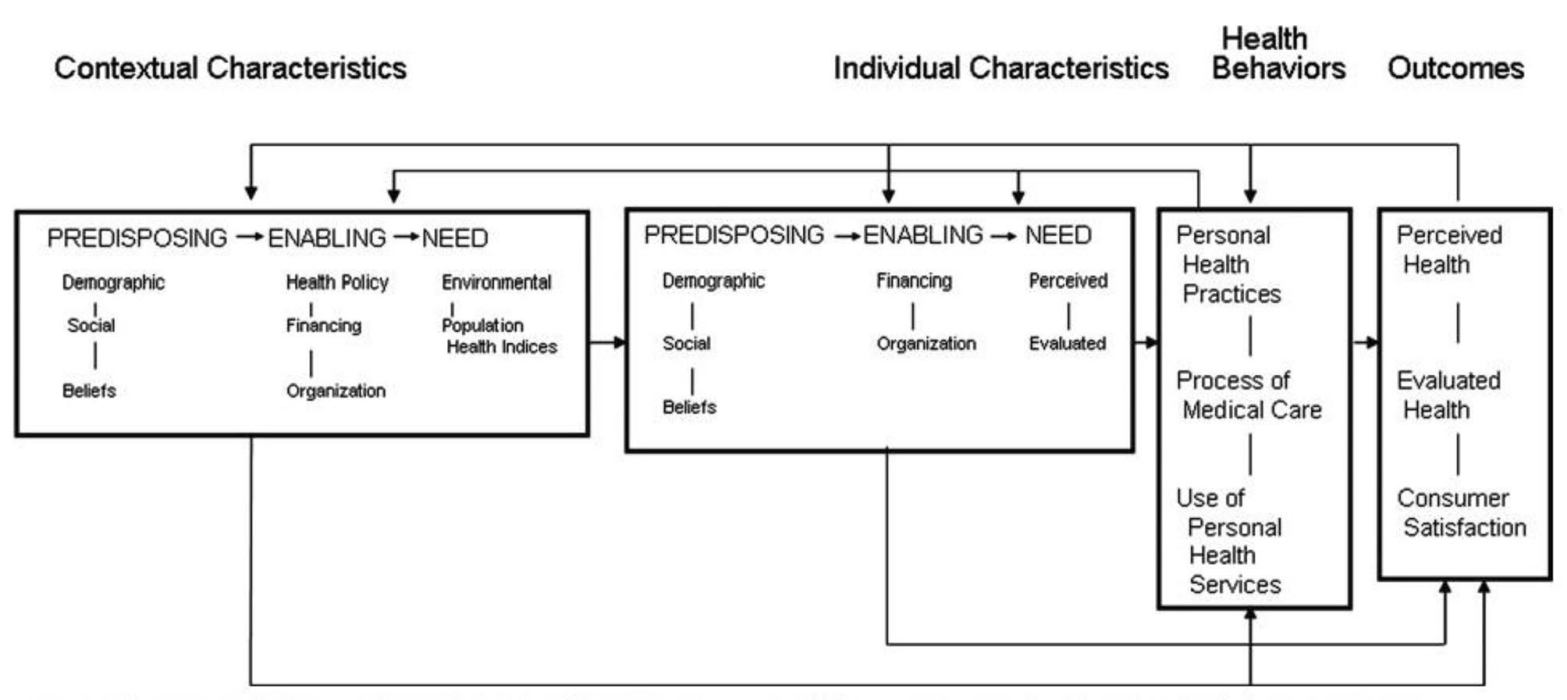
Phase 5: A behavioral model of health services use including contextual and individual characteristics

There has always been concern about why some individuals have good access to care and others do no [13–16]. National health surveys, which collect data at the individual or household level, have served multiple purposes for policymakers, providers, and researchers for more than 75 years. The behavioral model of “Health Services Use” is one of the most applied models serving to discover the underlying factors potentially affecting the utilization behavior of individuals. This model- named also as Andesen model- has been revised in response to the emerging issues in health policy and service delivery, peer review, critique of prototypes, and new developments in health service research and medical sociology. Revisions have generally been added to this model, but its core components or relationships have not changed significantly. As a result, Fig. 1, which shows the phase 5 of the model (the last phase) contains most of the components of the eralier models [14, 15].

The initial model was developed in the late 1960s to help the development of policies to promote equitable access, as well as understanding why only some families utilized health services. This was not the first or only model at the time, but it was an attempt to integrate a number of ideas about the “how” and “why” of utilizing health services. The basic model indicated that people’s use of health services could be a function of their desire (Predisposing Factors), the factors that led to or prevented the use (Enabling or Impeding Factors) for care, along with their needs (Need). In upcoming versions, the health care system was explicitly included to highlight the importance of national health policies. Also, health status, both perceived by the study population (perceived health or perceived need) and evaluated by experts (evaluated health or evaluated need), was added as an outcomes to the model [14]. The fourth phase included feedback loops, which showed that outcomes could affect the predisposing, enabling characteristics and needs of the population, as well. Finally, in phase 5, as the last stage of the model, there was emphasis on the fact that by focusing on individual determinants in the context of the community (contextual), understanding the health services utilization could be achieved in the best possible way [15]. In short, the final Andersen model (Phase 5) includes the contextual features of the community (predisposing, enabling and need factors), Individual characteristics (predisposing, enabling and need factors), health behaviors ( components of personal health measures, medical care process and utilization of personal health services) and outcomes (perceived health components, evaluated health, and satisfaction) [12, 15, 17, 18].

Definitely, all people have a right to receive medical care regardless of their ability to pay for the care; thus, equitable distribution of health services is a serious responsibility of health policy makers and administrators. Therefore, it is crucial that managers, health professionals and healthcare providers be fully aware of the predictors encouraging the use of dental services and reduce social injustice and inequality. Given that different studies have evaluated different indicators and factors related to the use of dental services, it is necessary to review these studies to find out these important factors as a comprehensive evidence-based guidance for evaluating the utilization of dental services [19]. Thus, this study aimed to analyze articles published between 1968 and 2020 on factors associated with dental services utilization among the general adult population.

## Materials and methods

This is a scoping literature review allowing the rapid mapping of the key concepts underpinning the research area of dental care utilization and the main sources and types of evidence available, to summarize and disseminate findings to policy makers who might otherwise lack time or resources to undertake such work themselves [20–22]. The study was based on the Arksey and O’Malley’s framework, which comprised five methodological steps [22] as follows:

### Steps1 and 2: identifying the research question and the relevant studies

The guiding question of this scoping review was:

What are the proposed determinants and predictors of dental care utilization based on the Andesen model?

The inclusion criteria included articles on those factors associated with the utilization of dental health services by adults 18 years old and above, published between 1968 and 2020, and available in English. The start date of 1968 was chosen because serious studies on the determinants of health service utilization appeared to have become relatively pervasive and focused since this date [14].There were no restrictions on the study design. The exclusion criteria were duplicate studies in databases and publications not fully available .

Keywords were selected under two main concepts: determinants of dental care utilization [with the main keywords but not confined to them; (“Facilities and Services Utilization” [MeSH] OR utilization OR use) AND (dental OR dentistry) AND (predictors OR determinants)], and the concept of the applied models [ Andesen Or “behavioral model”))]. Searches were conducted in some electronic databses including PubMed, Google Scholar and Scopus, and a combination of the keywords under the two main afore-mentioned concepts. (Search query for the PubMed is attached as Apendix I. The same combination of keywords were also used in the Scopus)

No specific databases were searched separately to elicit gray literature including theses and dissertations, research and committee reports, government reports, conference papers, and ongoing research. However, reference lists of the published articles on oral health utilization and behavioral models were checked to identify further relevant studies. The research question, and the search strategy were designed and discussed by both authors of the present study. AN searched, removed duplicated articles, matched the obtained papers containing eligibility criteria, and then extracted the data from the included papers. BT revised the results and interpreted the data.

### Step3: study selection

All the relevant articles were screened based on their title and abstracts; those reporting the utilization of dental care and its potential predictors among adult populations were chosen; these included original articles, online available dissertations, and official reports. In addition, articles were analyzed to find whether the conceptual model of Andesen has been applied or not.

No restrictions were considered regarding the terms of study time, or location. If the data set was common in two or more articles, only one was included. Finally, two researchers independently reviewed all included studies.

### Step 4: charting the data (data items and data charting process)

A data-charting form was developed to assess dental health utilization studies by the focus group discussion of a panel of four experts including two dental public health and two health policy specialists to make decisions on the key items needed to be extrapolated from the articles. The data charting form was pretested by five randomly selected articles, resulting in a satisfactory level of agreement between the authors. The items of the checklist included author, year, and place, sample size, study design, model type, oral health predictors or determinants and indices, statistical test, and outcome measure. The Andesen Behavioral Model was chosen as the basic and overwhelming model, and the determinant factors were then categorized as predisposing (demographics, beliefs and social structure), enabling (family and society) and need factors (perceived and evaluated). Utilization of dental care was considered as the outcome measure and its measuring criterion in different articles was reported.

## Step 5: collating, summarizing and reporting the results

The methodological quality of the retrieved articles was not formally appraised as we were to present and overview the elicited articles. In order to present a narrative account of the existing literature, we used a thematic constructing to illustrate our findings in two ways. First, attention was given to the basic numerical analysis of the extent, nature and distribution of the included studies. Using tables and charts mapping, we tried to group the studies based on their year of publication, geographic distribution, the range of included indices and the type of their measurement and analysis, to shed light on the dominant areas of research in terms of geographic area, measured criteria and the statistical analysis.

Second, a directed content analysis method was used to probe the comprehensiveness of the studies in terms of considering the determinant factors in different levels suggested by the Andesen model. Comprehensiveness was defined on whether at least one indicator of each level was reported in the included articles. Both of the authors investigate all of the studies and any disagreements were resolved through discussion.

## Results

The initial search was conducted in February, 2020, resulting in 927 potentially relevant articles. After omitting the duplications and relevance screening, 263 citations met the eligibility criteria based on title and abstract, and the corresponding full-text articles were procured for review. After updated search in September, 2020, and data characterization of the full-text articles, finally, 52 articles were included in the analysis. Six articles were written based on three common Surveys that just three of them were considered (Appendix II).

**Fig. 2:**
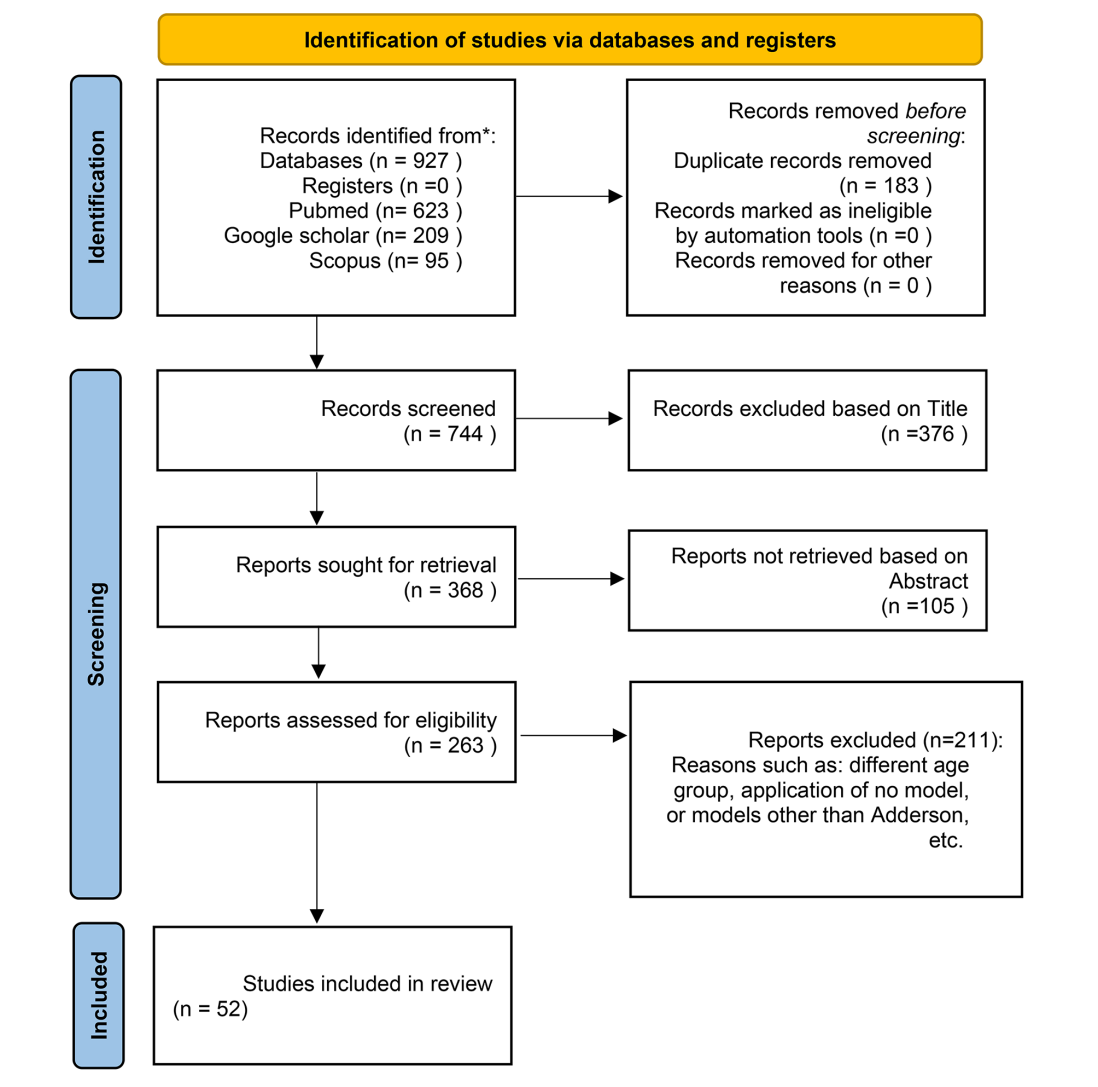
PRISMA flow diagram of the included articles

**Fig. 3:**
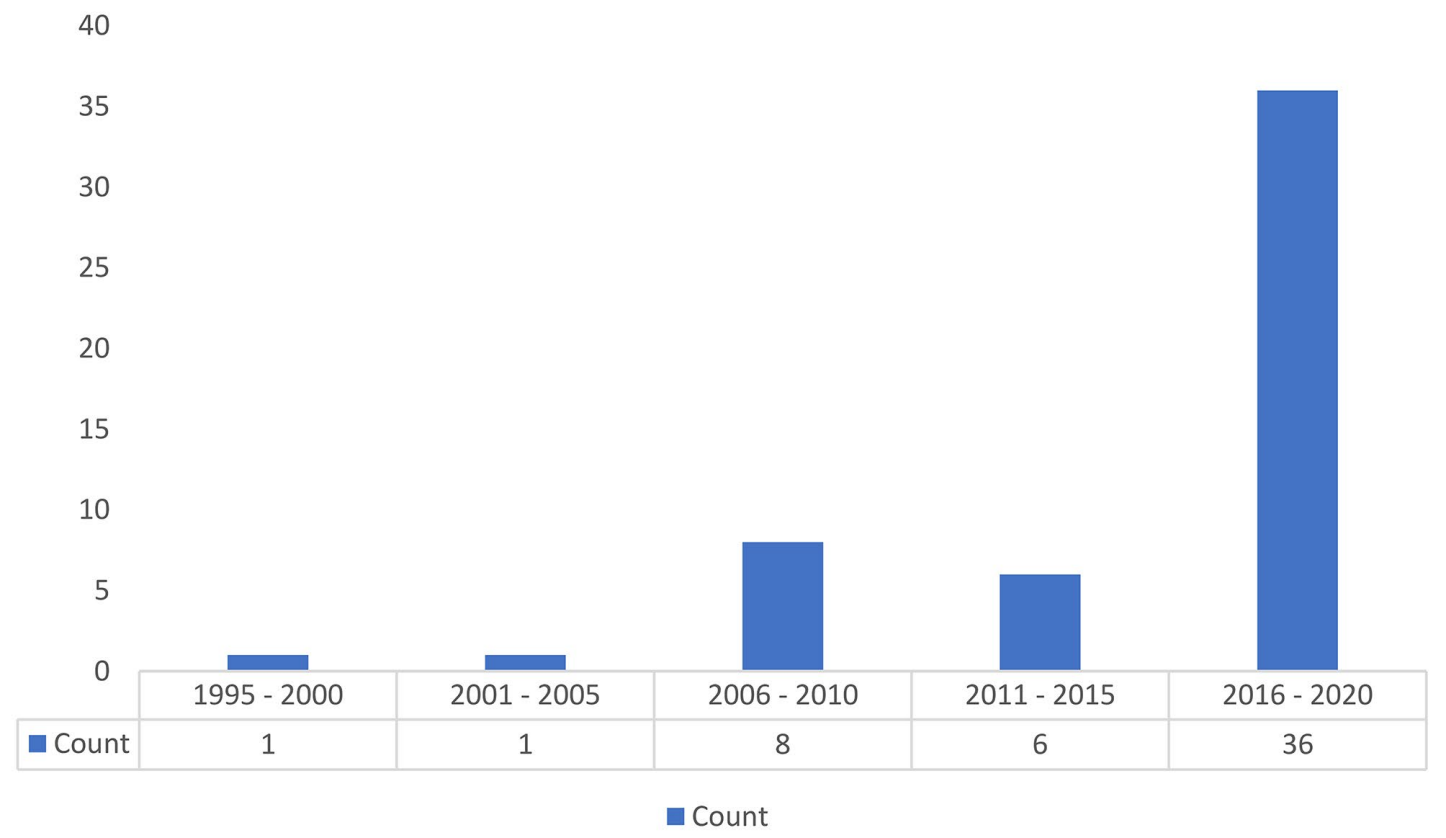
Frequency of published articles by year

**Fig. 4:**
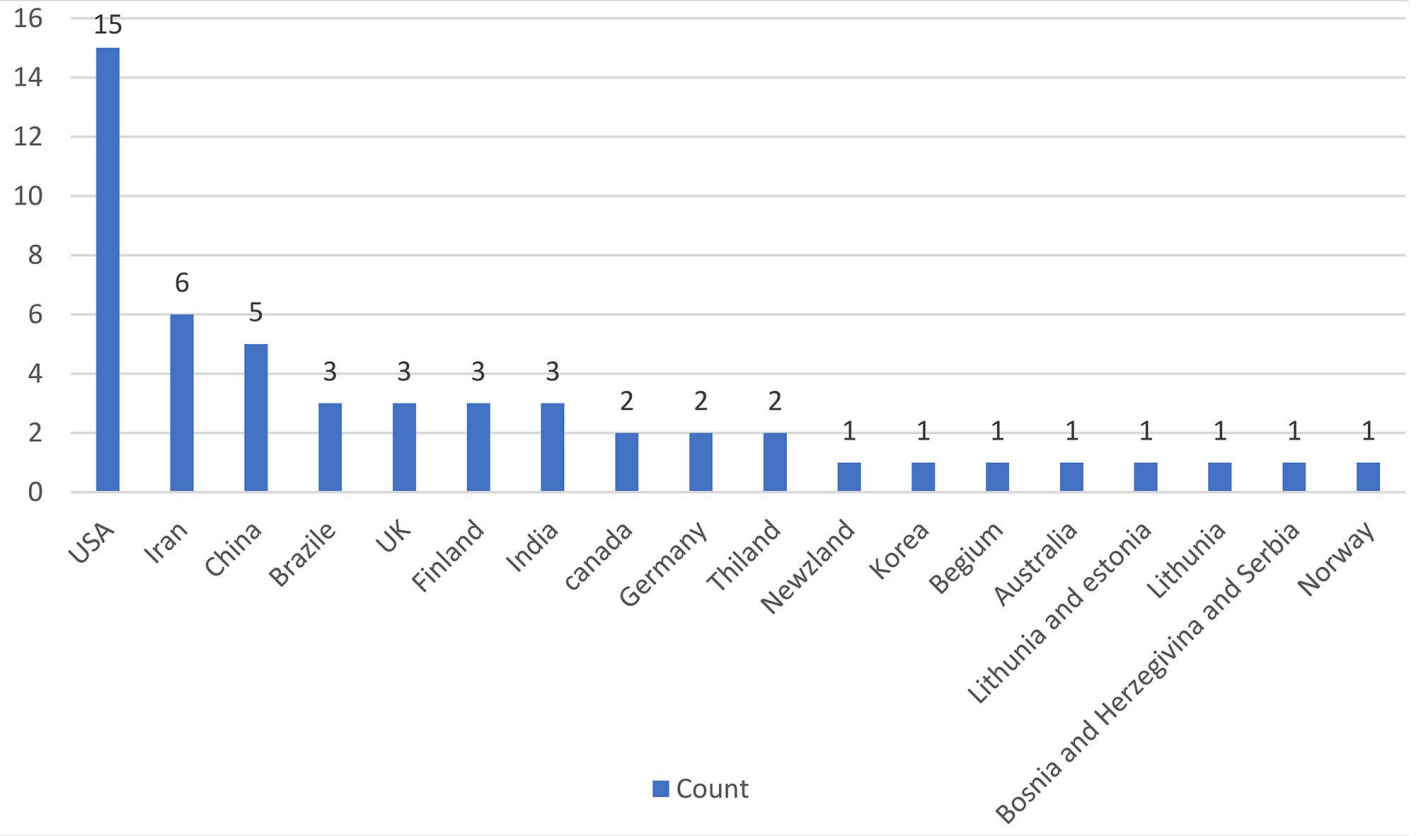
Frequency of published articles in each country

The flow of articles, from identification to final inclusion, is represented in Fig. 2. 36, out of the 52 articles (69%), had been published between 2016 and 2020. Figure 3 shows the distribution of all dental care utilization studies elicited in this study based on the year of publication. The frequency of countries that had studied dental services use in adults is shown in Fig. 4. The USA had conducted the most research (n = 15) in this scope.

**Fig. 5:**
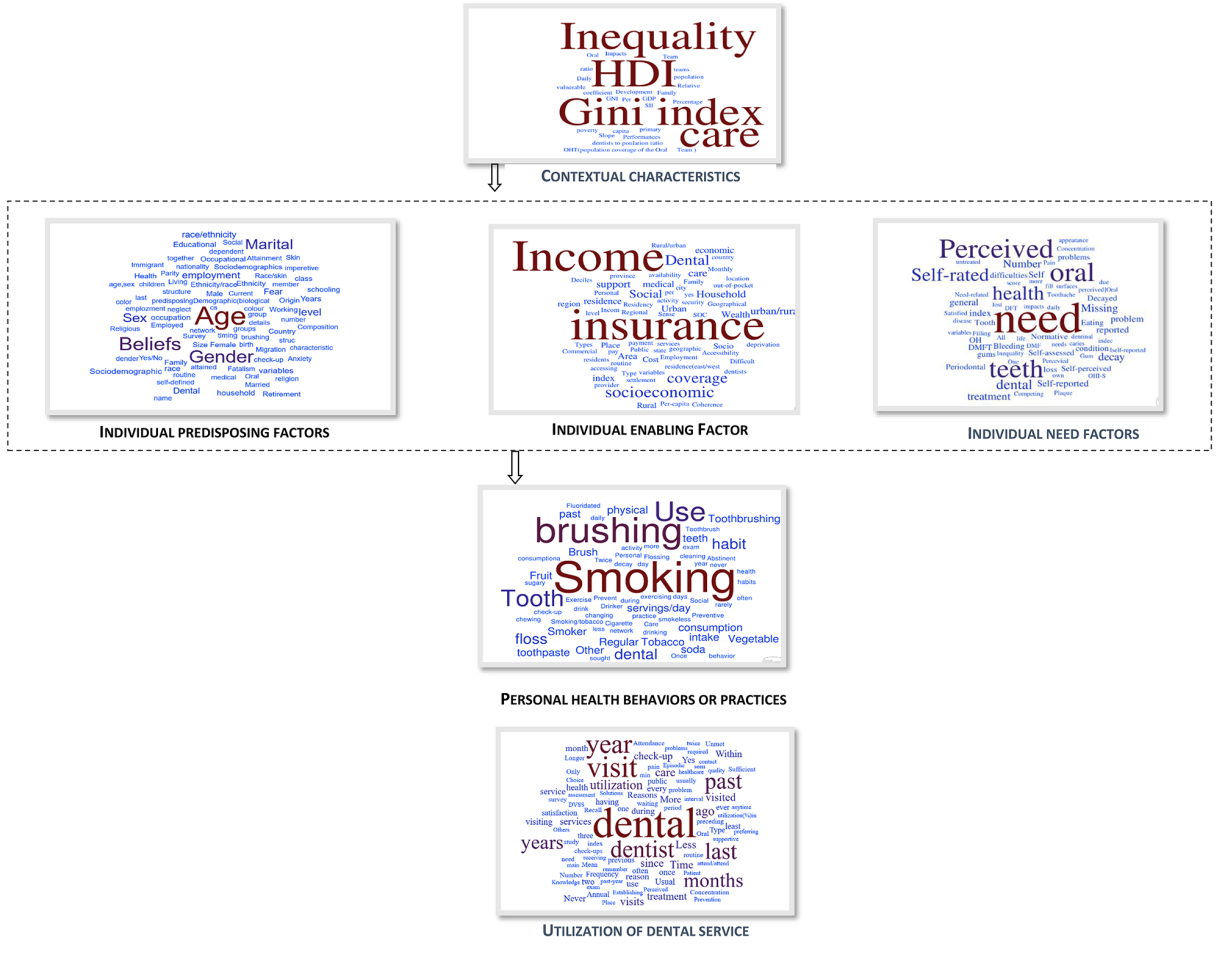
The high-frequent determinants in each domain visualized by world cloud diagrams

According to the Andersen model, studies were expected to assess the predisposing factors, demographic characteristics (age, sex, marital status and past illness), social structure (education, race, occupation, family size, ethnicity, religion and residential mobility), beliefs (values related to health and illness (e.g., self-reported oral condition and timing for routine check-ups)), attitudes toward health services (e.g., dental fear, dental anxiety, oral health attitude), and knowledge about diseases (e.g., score of oral health knowledge) [17]. The summary of the considered factors in the field of dental care utilization are presented in Table 1. The full results based on the proposed evidence table are demonstrated in Appendix III. Also, using the “Word it out” software (Available at: https://worditout.com/word-cloud/create) the high-frequent determinants in each domain are visualized in Fig. 5.

**Table 1:** Summary of the applied determinant factors in the dental care utilization literature based on the Andersen model

Determining factors based on the Andersen Model	Examples applied in the retrieved literature on dental care utilization
Contextual factors	dentists per 100,000 population in state of residence, Absolute income measures Gini coefficient, GDP, Relative Index of Inequality (RII), Slope Index of Inequality (SII), life expectancy of the city according to the component Longevity of the Human Development Index (HDI), the component Education of the HDI, the estimate of the population coverage of the Oral Health Team (OHT), HDI-Income, Per capita expenditure in primary care, Per capita expenditure in oral care, programs Family oral health teams coverage, Oral Impacts on Daily Performances, Percentage of extremely poor, Percentage of vulnerable to poverty
Individual predisposing factors	Demographic characteristic (gender, Age, Marital status, Race, Ethnicity, Skin color), Social structure (Education, employment status, Family Size, Retirement Status, household size, Immigrant status, Occupational class, Subjective social status, Participation in community meetings and associations, Number of household member ), Health Beliefs (Dental Fear, Fatalism, self-defined health status, Oral health awareness, Oral Health attitude, Dental neglect scale, Corah’s Dental Anxiety Scale (CDAS)
Individual enabling factors	Income, Insurance coverage, type of settlement, (urban/ rural), wealth index, socioeconomic characteristics (SES), Financial autonomy, Ratio of family income to poverty, annual household income, have a health care provider, Distance to the nearest dental clinic (km), Source of dental treatment, Dental cost in the past 12 months (CNY), Out-of-pocket payment (%), Method of dental Care payment, Frequency of Social support, Number of residents in the household, Number of rooms in the household, Registration in the primary care, Material circumstances, Sense of Coherence (SOC), ratio of inhabitants per dentist,
Individual need factors	self-reported need for various dental services, Normative need or evaluated need identified by the dental examiner, Self-rated dental appearance, Estimated value of lost productivity due to dental problems, Oral impacts on daily life,
Personal health behaviors or practices	Smoking status, physical activity, fruit and vegetable consumption, soda consumption, Tooth brushing behavior, Alcohol consumption, Having risky dietary habits, Use of dental floss, Use of tooth paste,
Utilization factors	Dental service use in the past 12 month, the main reason for their last visit, Patient satisfaction (DVSS = Dental Visit Satisfaction Scale), Dental visit during the past 3 years, last dental visit, Perceived quality of treatment received, the reasons for non-use of dental health-care

Regarding this approach, in the section related to individual predisposing factors, about 30% of studies (with at least one item) had mentioned all three domains of demographics, social structure and beliefs, simultaneously [3, 9, 12, 23–35]. 90% of the articles had considered the characteristics of the demographic and social structure in the study together, with or without belief characteristics (3, 5, 7–9, 11, 12, 16 and 23–63), and all 52 articles reported at least one item of demographic characteristics. Finally, 30.76% had considered the characteristics of beliefs simultaneously, with the two characteristics of demographics and social structure (3, 9, 12, 23–35).

The most prevalent indices used to assess the demographic characteristics were age [3, 5, 7–9, 11, 12, 16, 23, 24, 26–41, 43–50, 52, 53, 55, 57–69], gender [3, 5, 7–9, 12, 16, 23–33, 35–41, 43–50, 52, 55, 57–64, 66–69] and marital status [3, 5, 9, 11, 16, 23, 35, 36, 38, 40, 41, 43, 47, 49, 52, 53, 61, 63, 64, 69]. To examine the social structure, indicators such as education [3, 5, 7–9, 11, 12, 16, 23, 24, 26–41, 43, 44, 46, 48–50, 52, 53, 55, 57–63, 67, 69] and in one-third of cases, occupation [5, 8, 9, 16, 24, 26, 27, 32, 33, 38–40, 44, 46, 52, 58, 65] were used. Indices including attitudes toward oral health services (prevalent indices; dental fear survey and dental anxiety scale [12, 25, 27–30]) and values for health and illness (common index; self-reported oral condition or status [3, 9, 23–26, 33–35]) were used to assess the belief component.

For the evaluation of enabling factors, Andersen et al. have considered two factors of family [17] or financing [15] (income, health insurance, type of regular source, payer type including commercial insurance, Medicaid, Medicare, self-pay, etc., and access to regular source) and the community [17] or organization [15](ratios of health personnel and facilities to the population, price of health services, region of the country (place of residence, district of residency, regional level, accessibility to health clinic, distance to the nearest health clinic (km), and urban-rural character).

Accordingly, in 84.61% and 59.61% of studies, income levels [3, 5, 7–9, 11, 12, 16, 23, 24, 26–33, 35–37, 40, 41, 43, 45–48, 50, 52, 53, 55, 57–65, 67–69] and insurance feature [3, 7–9, 11, 16, 24, 29, 31, 33, 35–37, 40, 41, 43, 45, 47, 49, 53, 55, 58, 60–62, 64–66, 68, 69] were assessed, respectively. 21% of the studies had considered urban/rural status, 21.15% had assessed the region of country, and other features including the of type of regular source, access to regular source and ratios of health personnel and facilities to population were reported infrequently in less than 12% of studies [5, 7–9, 12, 26, 30–32, 36, 40, 43, 45, 47, 50, 53, 55, 61–64, 66, 68, 69].

According to the Andersen model, the need factor is divided into two parts: perceived need (disability (e.g., chronic painful dental ulcers), symptoms (e.g., difficulties in eating food, difficulties in chewing/biting foods, experiencing toothache, painful gums, feeling tense, feeling embarrassed), diagnoses (e.g., self-perceived oral health status, selfreported oral health problem) and evaluated need (symptoms, diagnoses (e.g., periodontal disease status, gums or gingival diseases, number of teeth present, number of untreated dentinal decay, etc.).

Accordingly, 57.69% of the retrieved studies had considered the perceived need features [3, 7, 8, 11, 12, 16, 23–27, 29–32, 34, 35, 38, 40, 41, 48–50, 52, 55, 61, 65–67, 69] and 38.46% took into account the evaluated need features [3, 16, 23, 25, 27, 29–31, 33, 35, 39, 41, 44, 48, 49, 53, 55, 57, 63, 67]. The scales to assess these two features were mostly general state (self-perceived oral health status) and diagnosis (number of missing teeth, number of present teeth, number of decay teeth and DMFT) for the perceived and evaluated need, respectively.

Factors of personal health behaviors (such as smoking status, frequency of brushing, drinking alcohol, physical activity, soda consumption, tobacco use, use of dental floss, eating healthy food, fruits and vegetables, use of toothpaste, etc.) were examined in 42.30% of the studies with at least one item [5, 8, 9, 23–26, 29–33, 35–39, 57, 61, 64–66, 68]. The most prevalent indices used in the studies were smoking status [5, 8, 9, 23, 26, 35–39, 57, 66], frequency of brushing [24–26, 30–33, 39, 57, 61, 64], drinking alcohol [26, 29, 35, 37–39, 57, 65, 66], physical activity [5, 29, 35, 36, 65] and smokeless tobacco use [8, 26, 57, 66].

### Outcome measures

The main outcome measures, based on the Andesen model, were expected to be utilization of health services and satisfaction. In the retrieved studies, dental services utilization was assessed by six types of questions, including the dental visit during the past one month, the past six months, the past 6 to 12 months, less than 12 months, the past 12 months, and more than the past 12 months. The dental services utilization in terms of the last visit during the “past 12 months” was assessed more commonly in 78.84% of studies [3, 5, 7–9, 11, 23–25, 27–37, 39, 40, 45–50, 55, 58–66, 68, 69]. ‘More than the past 12 months’ was reported as the outcome measure in 34.61% of the studies [3, 7, 9, 11, 12, 16, 26, 30, 33, 34, 37, 41, 45, 46, 55, 58, 63, 67]. Other categories were reported sporadically. For example ‘less than one year’ was found in five studies [7, 11, 33, 41, 46], ‘between 6 and 12 months’ in two studies [3, 63], ‘the past 6 months’ in three studies [24, 38, 63], and ‘the last month’ in only one study [43]. Satisfaction with the received dental care was considered as the outcome measure less frequently [23, 26, 29, 35].

### Contextual factors

As previously mentioned, Andersen et al., in the phase 5 of their model, divided the main sections of community contextual characteristics in the same way that individual characteristics have been traditionally divided; these included the predisposing factors (demographic, social and beliefs), enabling factors (health policy, financing and organization), and need characteristics of the population and their use of health services (environmental ((including physical, political, and economic components, not assessed in this study) and population health indices (perceived need and evaluated need)) [15]. Only 11.54% of the studies had evaluated the contextual characteristics [3, 9, 44, 55, 67, 70]; half of them considered the characteristics of the contextual, predisposing demographic and social characteristics (e.g., HDI (Human Development Index)-Longevity, HDI-Life expectancy, HDI-Education and Relative Index of Inequality (RII) and the Slope Index of Inequality (SII)) [44, 55, 67]. Four out of these 6 articles had addressed contextual enabling factors [3, 9, 55, 67, 70], of which three ones referred to contextual enabling health policy (such as active dentist per a 100,000 population, family oral health teams coverage) [9, 55, 67] and four contextual enabling financing (e.g., HDI-Income, Economic city level, GDP and GNI per capita) [9, 55, 67, 70]. Thirty three%(2 out of 6) of these articles had mentioned the need factor [55, 70]; one of the articles reported the contextual perceived need (Oral Impacts on Daily Performances, Dental pain) [55] and both reported the contextual evaluated need (DFMT index and Components, Need for denture) [55, 70]. Consequently the most prevalent predictors to assess contextual characteristics were enabling financing (HDI-Income, GDP per capita) [3, 55, 67, 70] and predisposing (HID-Longevity, HDI-Education) [44, 55, 67] and following these, enabling health policy (Family oral health teams coverage) [9, 55, 67].

#### Some miscellaneous points

Some studies had divided the utilization factors in different ways; for example, in the study done by Deguchi et al., race, education and income had been included among demographic characteristics [36]. Another study applied marital status as a subset of social structure and instead of the demographic factor, the term biological imperative, which included age, gender and race/ethnicity [23], was considered. Also, Lutfiyya M.N. et al. have placed race/ethnicity and geographic location in the demographic category [9]. Meanwhile, some other studies have combined demographic and social indicators and presented them under the socio-demographic title [11, 32, 38, 39]. Although, there have been studies that had applied different categories, they still used the important indicators in their research [48, 50].

#### Comprehensiveness

We evaluated the articles in terms of comprehensiveness based on the number of domains included; articles that had all six Contextual, Individual predisposing, Individual enabling, Individual need, Personal health behaviors or practices, and dental services utilization factors (at least one item in each factor) were considered as quite comprehensive ones. Those with four or five factors were considered as relatively comprehensive, and the less were deemed as not comprehensive. Accordingly, 71.15% of articles were relatively comprehensive (3, 5, 7–9, 11, 12, 16, 23–27, 29–33, 35–41, 45, 48–50, 55, 57, 61, 63, 65–67 and 69) and 28.85% were not (28, 34, 43, 44, 46, 47, 52, 53, 58–60, 62, 64, 68 and 70) .

## Discussion

Dental-service utilization is an important factor known to be associated with better dental care (i.e., early screening and treatment of dental diseases). To obtain a clearer understanding of the influence of contextual and individual factors on dental-service utilization, we evaluated the related studies based on the Andersen model and elicited the factors usually considered in these studies. In following, the proposed mechanisms and ways of effects of these factors on the utilization of dental care among different populations will be discussed more thoroughly;

### Individual predisposing, demographic characteristics (age, gender and marital status)

#### Age

It has been previously shown that there are significant associations between age and oral health care utilization [5]. However, the direction of this association can differ, depending on participants characteristics in the study. In some studies, it has been reported that the young age group has more dental visits than the older one [5, 9, 43, 48, 55, 68, 69]. However, the definition and range of the young age group were different in the published articles, such as 18–24 [5], 18–44 [9], 15–45 [43], 20–34 [48], 18–35 [68], 18–44 [55]) and the older age group (≥ 65 [5, 9], ≥ 66 [43], 50–64 [48], > 65 [68], ≥ 65 [55], 60–74 and ≥ 75 [69] [43]).

Siljak s. et al., 2019, in the Republic of Srpska (RS), found that the youngest age group of participants (18–24 years old) had the highest frequency of dental visits (38.8%), while the lowest (9.8%) was recorded for the oldest participants (≥ 65 years old). In other words, with the rise of age, dental visits decreased, which could be partly explained by the poor awareness of the oldest of the importance of periodic dental visits for the prevention and effective treatment and also, insufficient financial resources as a middle-income (developing) country [5]. Also, in a study conducted by Lutfiyya N.M. et al. (2019), in the USA, the youngest age group of participants (18–44 years) had the highest (45.4%) frequency of dental visits in the past 12 months, while the lowest (20.3%) was, again, found among the oldest participants (≥ 65 years old). To explain the reasons, the majority of the oldest people faced a higher socioeconomic burden and less education and therefore, had greater odds of not having seen a dentist in the past 12 months [9]. In agreement with other studies, Rezaei S. et al., in 2019, showed that dental care utilization was the lowest among the oldest Iranian age group (66 and above), which could be justified by the fact that dental care services were not fully covered by helth insurance in Iran and households should pay high out-of-pocket to receive dental care services. Thus, the SES of households and pro-rich inequality in dental care utilization can be partially explained by the affordability of dental treatment costs that might be compromised by age [43]. Although oral health problems increase with age, the odds of visiting a dentist were the lowest for the oldest age group of Estonians and Lithuanians [50–64], even after adjustment for oral health indicators (including edentulism). Therefore, the high cost of services might be a more likely explanation [48].

The positive change values for the concentration index (to quantify the degree of inequality) for dental care utilization among Chinese older people (60–74 years and ≥ 75) were larger than those among the middle-aged adults (45 to 59 years), thus indicating that the older people not only had a lower likelihood of using dental care services than the middle-aged adults, but also used dental services less often. There are two possible reasons for this difference in change. Firstly, older people may have less income and be more sensitive to the price of dental care than the middle-aged adults. Most of dental treatment is unaffordable for the poor old people. Secondly, mobility disability and function limitations may create barriers for older people to access dental services.

However, there were some other studies revealing that the old age group had more dental visits than the young one [23, 32, 36, 37, 64, 66]. Again, the definition of this age group was varied among different studies, which included age groups ≥ 65 years [23, 36], ≥ 80 years [37], not mentioned exactly [65–85 and ≥ 85] [64], > 30 years [32], and 45–64 and ≥ 65 years [66]), which had more dental visits than a young age group ( 25–44 years [36], 30–44 years [23], 60–69 years [37], not mentioned exactly [18–44] [64], 18–30 years [32], 18–44 years [66]).

Herkrath F.J. et al. (2018), in Brazil [55], showed the odds of visiting a dentist more than 12 months ago were significantly higher for the older adults (above 65 with OR = 2.91), as compared with the young (OR = 1.22) (18–44 years). In a study conducted among Indian black men, predisposing factors such as older age (65 + year 48.21, 45–64 years 46.84%) were positively associated with past year dental utilization. Generally, it has been suggested that being older (especially with being married and having higher levels of education) may be associated with greater levels of social interaction, social participation, and overall higher life satisfaction [66]. Also, Rezaei S, et al. indicated that older age was positively associated with the utilization of both general and dental care. It was found that the proportion of individuals ≥ 50 years of age who had visited a dentist was 12% higher than that for people < 30 years of age; it was explained by the fact that health is a capital good; accordingly, as aging increases, health will depreciate at a certain rate. Thus, to maintain health, utilization of health services increases with aging. Generally, according to the Grossman Model on age, not only the demand for dental services, but also the demand for all health care is U-shaped. At birth, the demand for health services is high and declines as people enter the middle age. Then, the demand for health services increases [24].

#### Gender

It has been indicated that female participants were more likely than the male ones to visit a dentist [5, 7, 16, 29, 36, 39, 41, 47, 48, 60, 62–65, 67]. This can be explained partly by the evidence that women with a higher level of education and working may have both more health awareness and sufficient financial ability, appreciating the importance of regular dental visits. Also, it might be explained by different norms in help-seeking behaviors in men and women, especially at the same socioeconomic status level [5, 39, 48]. In a study conducted by Muirhead in Canada [41], it was revealed that male working poor persons were more likely than their female counterparts to have not visited the dentist within the past year, even after adjusting for enabling resources and need factors. Their findings were explained partly by the fact that, in the context of working poverty, male working poor persons often work longer, are more unsociable and have less flexible hours than females, which could limit their ability to access dental care outside the normal workday [41]. However, Gupta A. et al., in 2019, reported that young, Mexican-American, and other minority race-ethnicities women were more likely to have never visited a dental clinic. These women reported that the dental problem would “go away” as the main reason for not visiting a dentist [11]. In addition, Kim Ch. et al. found that gender did not show any significant relationships with using dental care (except for examination) [59].

#### Marital status

In some of the studies, it was found that utilization of dental services was significantly associated with social relationships (marital status / living with partner or spouse / cohabitation status) [16, 36, 43, 47, 59, 63, 65]. Being married or living together with a partner or spouse was found to enhance the chance of utilizing dental check-ups [47]. Also, Kim Ch, et al., 2015, indicated that oral health utilization for examination was significantly higher among individuals who were living with their spouse [59]. Those cohabiting (22%) reported admitting for preventive check-up more often than their counterparts did. However, Brzoska P. et al., in 2017, reported that only small differences could be observed in dental care utilization behavior based on marital status [64].

### Individual predisposing, social characteristics (education, occupation, ethnicity and race)

#### Education

In the study done by Siljak S, et al., in 2018, it was reported that persons with a low and middle level of education were approximately 70% and 50% less likely to attend a dental visit in the last year, as compared with those with a high level of education [5], which was in accordance with other studies [8, 9, 11, 24, 31–33, 36, 37, 39, 43, 44, 48, 55, 59, 65, 67]. This could be justified by the evidence that individuals with a higher level of education may have a greater health literacy, awareness of or interest in the importance of habitual dental visits [5, 31, 33, 39]. Schroeder S, et al., in 2018, also reported that lower educational attainment had been cited in the literature as one of the variables correlated to lower dental care utilization. On the other hand, the odds and likelihood of utilization of denture services were increased significantly in groups with a higher education level. The results of the elicited evidence also revealed a statistically significant relationship between the level of education and the frequency of visiting a dentist. In other words, individuals with a higher educational level tended to have a healthy lifestyle by seeking treatment for their dental problems at an earlier stage [24].

However, Chen M. et al., in 2019, found that education was not a significant predictor for regular attendance (39% = High School diploma/GED, 54% > High School diploma/GED) [12].There was also no significant association between the level of education and utilization of oral health services in the study done by Bommireddy V.S. et al., in 2016 [26].

#### Occupation

The elicited studies reported that the employed people had higher rates of regular attendance [39, 65]. In Iran, it was shown that having a higher paid job and a higher level of education might increase the chance of having commercial insurance coverage, which could have a strong impact on dental attendance [60]. However, there was also a study indicating that economic activity status and income level did not show any significant relationships [59].

#### Ethnicity and race

Based on the available evidence, it seems that the likelihood of non-utilization of dental services has been lower among adults with brown skin color (Ethnicity) in countries such as Brazil [67]. Also, Mexican-American and other minority race-ethnicities were independently more likely to have never visited a dental clinic [11].

### Individual predisposing, beliefs characteristics

#### Values and beliefs

Regarding predisposing factors, some of the studies found that oral health beliefs could affect oral health service utilization, especially in adults aged 35-44 years [62]. Dental fear is one of the beliefs that could cause non-habitual dental attendance and decreasing dental fear increases habitual attendance [28]. In the study done by Xu M. et al. among Chinese population, it was revealed that self-perceived oral health status (very poor/ poor/moderate/good/very good) was associated with oral health utilization [62].

### Individual enabling characteristics

#### Financing: (income, Health insurance)

Studies indicated that while lower-income older adults and those without insurance reported a higher proportion of need for dental care, older adults with higher income and those privately insured usually had a higher odd of utilizing dental care. Also, it is reported that those with higher income reported a greater proportion of need for teeth to be filled or replaced in comparison with middle- or lower-income individuals. Additionally, those with lower income and lower dental visit rates expressed a need for their teeth to be extracted. That might be explained by the fact that tooth extraction could be more affordable and does not require high cost and future monitoring [8].

Across some parts of the USA, greater dental utilization over a 3-year time period was associated with having dental insurance [12]. It has been indicated that persons in the lowest tertial of wealth utilized dental services 21% less frequently when compared with those in the highest one. In other words, chances for dental services utilization increased consistently with higher socioeconomic status [5, 36, 47, 60, 62, 69]. Therefore, it would be expected that expanding insurance coverage for dental care might reduce racial-ethnic, educational, and economic disparities in dental care access and the unmet need for dental care [11]. However, in contrast to other findings, Herkrath F.J. et al., in 2018, in Brazil, indicated that, odds of visiting a dentist over 12 months ago were significantly higher for older adults who were male, with brown race/skin color, low schooling, low social networks, low income, with no health insurance, poor perceived dental needs and higher number of missing teeth [55].

#### Organization: (region of country, Urban-rural character, access and availability)

Siljak S. et al., 2019, reported that urban residents had a higher likelihood of visiting a dentist than those who lived in rural areas [5]. Also, state residency was the most effective predictor of dental services utilization among adults in north-central Appalachia, according to Chen M. et al.’s 2019 study [12]. It has been demonstrated that there are distinct regional variances in the utilization of dental services for examination throughout the areas of Korea and that these regional variations were independent of individual-level socioeconomic considerations. Korean individuals were more likely to use dental services for oral examination in areas without severe regional deprivation than in those with the severe one, thus showing that context could affect dental care utilization for examination [59]. Several studies had also revealed that the use of dental services was less common in the rural areas [5, 7, 12, 26, 47, 49, 59]. Financial constraints were a significant factor in how dental services were used in rural locations and utilization of dental services was linked to their availability through primary care [7]. Expanding coverage, offering prevention services, and bolstering oral health education could all help to improve access to dental care among low-income populations because poor oral health in adults could have significant adverse impacts on general health [49].

### Individual characteristics: need

#### Perceived need characteristic

Several studies have suggested the increased probability of dental care utilization among people with lower self-rated oral health [5]. Chinese adults with worse self- perceived oral health status were more likely to use dental care as a result of a symptom-driven or treatment-oriented pattern. This pattern of usage was quite different from that of the tendency in high-income countries for regular dental visits which helped to prevent disease development and promoted oral health [62]. Additionally, people dissatisfied with their oral health were less likely to have visited the dentist in the previous 12 months than those who were satisfied (57 versus 25%) [23]. According to Muirhead VE. et al., there was no correlation between toothaches or oral pain and dental care-seeking behaviors, despite prior studies citing oral pain as a major motivating factor for seeking dental utilization. Instead, compared to other measures of oral health status, self-perceptions of oral health and the presence of a functional dentition were better predictors of service utilization. When compared to people without a clear-cut perceived need, working poor people with a perceived need for dental care were nearly three times more likely to visit the dentist in the past year [41].

#### Evaluated need characteristic

The evaluated need has been reported by the number of missing teeth or decayed teeth or the DMFT index [27, 29, 30, 44, 53, 55, 57, 67]. Evaluated need, which helps to identify a high-risk population or untreated dental problems, can be used as a measure of success in dental care delivery and outcome [25, 49, 67].

### Contextual characteristics

Regarding the contextual characteristics, although there were very limited studies assessing their effects on the dental care seeking behaviors, it was reported that adults living in cities with high HDI-income were 67% less likely to have had no dental visit than those living in the cities in the higher tertial. In a study, findings from multilevel mixed-effects linear models showed that participants residing in megacities with higher GDP per capita had more frequent dental visits after adjusting demographic characteristics, socioeconomic status, health status, health behavior and attitude, and oral health indicators [36].

## Conclusion

To conclude, these findings suggest that firstly, the dental care utilization behavior of people is a complex phenomenon and without an in-depth understanding of the multiple social-environmental, individual, sociodemographic and dental needs characteristics, it might be difficult to predict this behavior thoroughly. Secondly, it seems that in most of the study, it has been difficult to consider all factors simultaneously. In order to discover the conceptual linkages and feedback loops of the model, conducting more comprehensive future research seems to be necessary.

### Electronic supplementary material

Below is the link to the electronic supplementary material.

Supplementary Material 1

## Data Availability

The datasets analyzed during this study are included as the Appendix file.
