# Supplementary material for: Utilization of dental care among adult populations: a scoping review of applied models
Source: BMC Oral Health. 2023 Aug 27;23:596. doi: 10.1186/s12903-023-03323-1 (PMC10463392; doi:10.1186/s12903-023-03323-1)
Supplement: Supplementary file 1 — Supplementary Material 1 [file 12903_2023_3323_MOESM1_ESM.docx]

**Appendix I. PubMed search query**

((((((""dental visit"" OR access OR status OR satisfaction))) AND ((""Dental Care""[Mesh]) OR dentistry OR dental))) AND ((""Facilities and Services Utilization""[Mesh] OR utilization OR use))) AND ((predictors OR determinants OR factors))) AND model" =623

**Expanded search query in PubMed Jan 2021**

""utilizers""[All Fields] OR ""utilizes""[All Fields] OR ""utilizing""[All Fields] OR (""statistics and numerical data""[MeSH Subheading] OR (""statistics""[All Fields] AND ""numerical""[All Fields] AND ""data""[All Fields]) OR ""statistics and numerical data""[All Fields] OR ""use""[All Fields])) AND (""dental health services""[MeSH Terms] OR (""dental""[All Fields] AND ""health""[All Fields] AND ""services""[All Fields]) OR ""dental health services""[All Fields] OR ""dental""[All Fields] OR ""dentally""[All Fields] OR ""dentals""[All Fields] OR (""dentistry""[MeSH Terms] OR ""dentistry""[All Fields] OR ""dentistry s""[All Fields])) AND (""predictor""[All Fields] OR ""predictors""[All Fields] OR (""analysis""[MeSH Subheading] OR ""analysis""[All Fields] OR ""determination""[All Fields] OR ""determinant""[All Fields] OR ""determinants""[All Fields] OR ""determinate""[All Fields] OR ""determinated""[All Fields] OR ""determinates""[All Fields] OR ""determinating""[All Fields] OR ""determinations""[All Fields] OR ""determine""[All Fields] OR ""determined""[All Fields] OR ""determines""[All Fields] OR ""determining""[All Fields])) AND (((""dental health services""[MeSH Terms] OR (""dental""[All Fields] AND ""health""[All Fields] AND ""services""[All Fields]) OR ""dental health services""[All Fields] OR ""dental""[All Fields] OR ""dentally""[All Fields] OR ""dentals""[All Fields]) AND (""access""[All Fields] OR ""accessed""[All Fields] OR ""accesses""[All Fields] OR ""accessibilities""[All Fields] OR ""accessibility""[All Fields] OR ""accessible""[All Fields] OR ""accessing""[All Fields])) OR ((""dental health services""[MeSH Terms] OR (""dental""[All Fields] AND ""health""[All Fields] AND ""services""[All Fields]) OR ""dental health services""[All Fields] OR ""dental""[All Fields] OR ""dentally""[All Fields] OR ""dentals""[All Fields]) AND (""visit""[All Fields] OR ""visitation""[All Fields] OR ""visitations""[All Fields] OR ""visited""[All Fields] OR ""visiting""[All Fields] OR ""visits""[All Fields]))) AND (""model""[All Fields] OR ""model s""[All Fields] OR ""modeled""[All Fields] OR ""modeler""[All Fields] OR ""modeler s""[All Fields] OR ""modelers""[All Fields] OR ""modeling""[All Fields] OR ""modelings""[All Fields] OR ""modelization""[All Fields] OR ""modelizations""[All Fields] OR ""modelize""[All Fields] OR ""modelized""[All Fields] OR ""modelled""[All Fields] OR ""modeller""[All Fields] OR ""modellers""[All Fields] OR ""modelling""[All Fields] OR ""modellings""[All Fields] OR ""models""[All Fields])",

| **Article 1** | **Similar Article(omitted)** | **Shared Database** |
| --- | --- | --- |
| Socioeconomic inequalities in oral health in different  European welfare state regimes | Inequalities in oral impacts and  welfare regimes: analysis of 21  European countries | data from the Eurobarometer  72.3, a survey carried out in 2009 |
| Oral health behaviour in migrant and non- migrant adults in Germany: the utilization  of regular dental check-ups | enabling and Predisposing Factors  for the Utilization of Preventive  Dental health care in Migrants and  non-Migrants in Germany | data from the cross-sectional survey ‘German Health Update 2010’ conducted by the Robert  Koch Institute |
| Impact of the Health Resources and Services Administration’s state oral  health workforce grant program on dental workforce density and access to oral health care | Impact of HRSA’s State Oral Health Workforce Grant Program on Dental Workforce Density and Access to Oral Health Care | data for 2006-2016 SOHW program awardees together with data from the 2016-2017 Area Health Resources Files (AHRF) and the 2016 Behavioral Risk Factor Surveillance  System (BRFSS) |

**Appendix II. Articles written based on similar data sets.**

**Appendix III**: evidence table of retrieved articles including the year of publication, place of the study, number of the participants, and determinant factors considered to assess the dental care utilization behavior

| **Comprehensiveness** | **Utilization**  **factors** | **Personal health behaviors or practices** | **Individual need**  **factors** | **Individual enabling**  **factors** | **Individual predisposing factors** | **Contextual factors** | **Number of participant/ study design** | **Author/year** |
| --- | --- | --- | --- | --- | --- | --- | --- | --- |
| Relatively | visited a dental professional or dental hygienist in the last year | smokeless tobacco use or chew ,  smoking status | self-reported need for various dental services(  for relief dental pain, need help to mouth problem, ..)  oral health status | Income,  Insurance coverage | Demographic (Age,  Social structure(  education,  employment, | ------ | 16829,  Historical cohort | 1.  Schroeder s. et al.  2019(5), USA |
| Relatively | utilisation of dental services in the past year,  the main reason for their last visit  the reasons for non-use of dental health-care services | smoking status,  physical activity,  fruit and vegetable consumption | - | type of settlement, (urban/ rural)  wealth index,  socioeconomic characteristics(SES) | Demographic characteristic cs( gender,  Age, marital status)  Social structure  (Education,  employment status ) | ------- | 4128,  cross-sectional survey | 2.  Siljak s. et al. 2019, european developing country |
| Relatively | Visited a dentist in past year? | smoking status,  soda consumption,  exercising in the past 30 days. | ------ | Income,  health coverage,  Financial autonomy  Rural/urban status | demographic variables  ( gender,  age,  marital status, race)  social structure (race,  Education) | ------ | 14564,  cross-sectional survey | 3.  [Deguchi](https://pubmed.ncbi.nlm.nih.gov/?term=Deguchi+M&cauthor_id=30861109) M. et al. 2019,  USA, Hawaii |
| Relatively | Have dental visit in the past 12 months? | Smoking – Cigarette use , | PERCEIVED Need  (Self-perceived oral health status)  Responses were recoded as a dichotomous variable representing satisfied (excellent, very good, or good) and not satisfied (fair or poor) (  Normative need or evaluated need  identified by the dental examiner. ((Seek care(immediat/routine,    Caries(0, 1, 2 and more)  Periodontal disease status(normal, mild, moderat to severe)  Teeth present (1-19, 20 and more)) | Ratio of family income to poverty, | predisposingDemographic(biological imperetive) ( age,  Gender, race/ethnicity  Marital status)  social structure  (Educational level, race/ethnicity , Marital status)  )  health beliefs  assessed by perceived general health  General health condition ( [A](Excellent/Very good/Good)  [B]( Fair/Poor) )  ) |  | 5806,  cross-sectional survey | 4.  Adunola F. et al. 2019, USA |
| Relatively | Dental visit “During the past 3 years, have you been to the dentist for routine check-ups or cleanings? | - | Dental Neglect Scale  DNS  (indicators about one's attitudes and values about investment in one‘s oral health) | Household income  Residency  (state residency) | Demographic  (age, sex)  Social structure  (Education  ,  Beliefs (Dental Fear Survey  Mean +- S,  Oral Health Fatalism  Mean +- SD  ) | - | 868  Historical cohort | 5.  Chen M. et al. 2019, USA |
| Relatively | a public denture service utilization over the past 5 years  health check-up during the past year or not  Public service utilization (vaccination)? | health check-up during the past year or not)  (not smoking or drinking alcohol(Neither), either or both smoking and drinking(Either/Both) | ------ | Economic condition,  working status  healthcare insurance recent illness | Demographic  (age,sex)  Social structure (education) | ------ | 38695  cross-sectional | 6.  Limpuangthip N. et al. 2019, Thailand |
| Relatively | last dental visit ?  [Within the past year,( Within Last 12 Months) ]  [ Within the past 2 years,  Within the past 5 years,  5 or more years ago, ( Longer Than 12 Months Ago) ] | smoking status, | --------  - | socio-  economic variables,  annual household income  have a health care provider  have a health care insurance  geographic location,( center,suburban, …) | Demographic  (sex, age, marital status )  Social structure (education attained, employment status, race/ethnicity  )  Beliefs (timing of last routine medical check-up, self-defined health status | Contextual enabeling resources:  [Health policy] (dentists per 100,000 population in state of residence )  (40.93–57.23 Per 100,000 ,  57.24–73.54 Per 100,000,  73.55–89.85 Per 100,000 ). | (weighted n=60,512,412)  Cross sectional survey | 7.  Lutfiyya M.N. et al. 2019, U.S.A. |
| Relatively | When was the last time you visited a dentist? 1- Never  2- I do not remember  3- More than 5 years ago  4- 3-5 years ago  5- 1-3 years ago  6- Less than one year ago  How often do you visit a dentist for check-up? visiting a dentist during 1 year ago | Brushing, | Evaluated need:  dentition status  ( All 32 teeth, Some decayed, Edentulous) | supplementary insurance  income rate$  Have you avoided or postponed visiting a dentist during 3 years ago because of its costs? | Demographic variables  (age, gender)  Social structure (education, employment)  Beliefs ( brushing) |  | 1185  Cross sectional study | 8.  Amiresmaili M.R. et al. 2018, IRAN |
| Relatively | Have you ever visited a dentist anytime in the past but not just past year  Yes / No  Where had you undergone the treatment  Reasons for preferring a particular centre Others advice  (satisfaction)  Perceived quality of treatment received | Frequency of brushing  Care sought    Frequency of changing tooth brush  Smoking/tobacco habits  Alcohol habit | Percevied need (diagnosis) :  Have you ever experienced dental problem  If yes, what was the most remembered episode of your dental problem | Income,  Socio economic status  Cleaning aid  Cleaning material | Demographic  ( name, age  ,gender)  Social structure  (education  ,religion, occupation )  Beliefs  -Reported barrier for not undergoing dental treatment inspite of experiencing dental problem  -Fear | ------ | 621  Cross sectional study | 9.  Bommireddy V.S. et al. 2016, India |
| Relatively | dental visit within the past 6 months. | Health-care behaviors and perceptions HIV care in last 6 months  Health-care  empowerment,  mean  Clinical measures  CES-D ≥ 16  GAD≥10 (  Viral Load below 200 copies/ml  CD4 count below 200 cells/ml (  #Risk behaviors  Alcohol use  Abstinent  0–7 drinks/week  > 7 drinks/week  Smoking status  Never  Current  Former  any substance use  (i.e., crack, cocaine, heroin, methamphetamine, illicit methadone, amphetamines, narcotics, hallucinogens, injected drugs, noninjected recreational drugs, nonmedical use of prescription drugs, marijuana/hash) | Oral health status :  Self-perceived fair or poor oral health  Yes/No | Dental insurance  Private  Ryan White  Medicaid/Medicare  None  New Southern WIHS site  Yes/No | Sociodemographics  Age (years),  mean  Married  Yes/No  Race/Ethnicity  White  Hispanic  Black  Other    Social structure  (Education  Less than high school  High school graduate  > High school graduate,    Employed  Yes/No | ----- | 1,442 women  cross-sectional survey | 10.Parish C.L. 2019, USA |
| Relatively | Annual attendance (AA, contact with the dentist at least once a year (at least three annual visits in a 5‐year period) | Toothbrushing frequency  Having risky dietary habits (caries and erosion)    Smoking habit | Evaluated need  (D3MFT,  No. untreated dentinal decay,  Restorative index,  DPSI  Dutch periodontal Screening Index ,  Number of teeth  Number of occluding pairs,  Mean Plaque Index ) |  | Sociodemographic  variables  (age  gender )  Social structure  (employment status  , education level ,  Country of birth,  Current nationality)  Beliefs ---- |  | 1340  Cross sectional study | 11. bottenberg P., 2019, Belgium |
| NO | utilization of dental checkups in the previous 12 months | ------ | ------ | SES  type of health insurance,  social support ,  the place of residence(east/west)  ,  the type of residence(urban/rural) | Demographic  sex  age  marital status | ------- | 41220  cross-sectional telephone surveys | 12.  Brzoska, P., 2017, Germany |
| Relatively | visit a dentist during pregnacy?  Frequency of visiting a dentist for a checkup | Frequency of brushing  Presence of own toothbrush  Use of toothpaste | [perceived]Oral health status  Self‑reported oral health problem  Oral health status  Self‑reported oral health problem | Household income (Rs. )  Distance to the nearest dental clinic (km)  Accessibility to dental clinic | sociodemographic details  (Age,  Ethnicity )  Social struc  (education level ,  Employment )  Beliefs (Oral health education received  Aware of connection between oral health and pregnancy ,  Aware of free dental services in government clinics  Importance of brushing  Importance of visiting a dentist |  | 300  Cross sectional study | 13. Barman D. et al 2019, India |
| Relatively | Have a dental use in  2 years period of study? | ------ | Perceived need (Health Status,  )  Evaluated teeth (Permanent Teeth  ) | Wealth Deciles  Family Income  Dental Coverage | Demographic  Age  Sex  Race  Marital Status  Social structure  (Household Composition,  Education ,  Family Size ,  Retirement Status )  Beliefs  ------ | ----- | 14970  Cross sectional study | 14. Manski R. J., et a. 2012, USA |
| Relatively | Frequency of visits to a dentist in the last year  Place for dental treatment  Visited the dentist in the past 12 months  (Yes,  No)  Visited dentist for check-up every 6 months  (Yes,  No) | Regular brushing  (Yes, No) | Self-rated oral health | health insurance coverage  income level per month  Source of dental treatment (Self-medication, Dentist, Herbal  treatment)  Cost of dental services for household | Demographic  age  gender  social structure  (level of education  employment status  household size)    beliefs  (Have a dental pain but do not visit dentist  Oral health as important as health elsewhere in the body | ------- | 1067  Cross-sectional study | 15.Rezaei S. et al. 2018(28), IRAN |
| Relatively | Reasons for not visiting a dentist in the past 12 months  the reasons for the last dental visit in the past 12 months | Frequency of teeth brushing  Use of dental floss  Tooth brushing habit | Perceived oral health status  had toothache or discomfort in the past 12 months (perceived need, symptom)  Perceived general health status  Evaluated need (DMFT (dmft)  Number of teeth with gingival bleeding,  Number of retained teeth  Unrepaired missing teeth  ) | Area  (Urban / Rural)  Annual household income per person  (low , middle, high)  Dental cost in the past 12 months (CNY)  Out-of-pocket payment (%) | Demographic (Age  Social structure  (Education  Beliefs  (Score of oral health knowledge  Score of oral health attitude | ------- | 172,425  Cross sectional survey | 16. Cheng M.L. et al. 2018, CHINA |
| Relatively | Type of treatment  Oral healthcare utilization(%)in 12 months preceding the survey  Conditional number of visits  Type of facility utilized | ------ | Self-assessed oral health | Living standards  [SES]  Geographic characteristics  ( region of urban/rural)  Insurance | Demographic  (age, dender,  Marital status  Social structure (  Educational level ,  Working status | ---- | 74057  Cross sectional survey | 17. Somkotra T. et ai. 2009, Thailand |
| NO | ---- | -------- | ---------- | ------- | ------------ | contextual  enabeling factors  Financing:  Absolute income measures  (Income  assessment)  GDP, $  GNI, $  Relative income measures  (income inequality assessment)  Gini coefficient  20:20 ratio  Contextual  Evaluated  NEED FACTORS  DMFT index and components  Care index:  Restorative index:  Treatment index: | 18 Rich Countries  An Ecological Study in Rich Countries | 18.  Bernabé E. et al. 2009, UK and PERU |
| Relatively | Episodic use of dental services  Mean months since last dental visit  Last year survey | Brush teeth less often than daily  Use dental floss ‘‘never’’ or ‘‘rarely’’ | Self-rated dental health  Self-rated dental appearance  Evaluated need :  OHI-S score  teeth missing due to caries (%)  DS decay surfaces  FS Filling furfaces  DFS(sd) | ----- | Demographic  (gender  Social structure  ------  Beliefs  DFS Dental fear scale  DNS  Dental neglect scale  (I keep up my home dental care  I receive the dental care I should  I need dental care, but I put it off  I brush as well as I should  I control snacking between meals as well as I should  I consider my dental health to be important | ----- | 1037  Cohort study  The Dunedin Multidisciplinary Health and Development Study (DMHDS) is a longitudinal study of a birth cohort of children who were born at the Queen Mary Hospital, Dunedin, New Zealand between 1 April 1972 and 31 March 1973 (4). The sample that formed the basis for the longitudinal study was 1037 children, and they were assessed within a month of their third birthdays. Periodic collections of health and developmental data (including dental examinations) have been undertaken since then, and the current study uses data collected at age 26. | 19.  Thomson WM. Et al. 2000, New Zealand |
| Relatively | Last dental visit  > =1 year  (more than one year)  the usual reason for dental visits. | ----- | Need;  1.Self-rated oral health  2.Functional dentition    3.Denture status  4.Toothache history    5.Oral health impact: pain  6.Oral health impact: chewing  7.Oral health impact: sleep    8.Oral health impact: work  9.Oral health impact: Discomfort/ embarrassment 11.Perceived need for treatment  Competing needs | Annual family income  Method of dental Care payment  History of inability to afford dental care | Sociodemographic  (Gender ,  Age ,  Marital status)  Social structure  (Education  , Parity: number of dependent children ,  Immigrant status  Beliefs ----- | ----- | 1049  A cross-sectional  telephone survey methodology | 20. Muirhead VE. et al. 2009, Canada |
| Relatively | Dentist visits in past 12 month  (Mean) | ------ | self-assessed general oral health status | Income  Insurance  employer-provided health benefits  province of residence  Social support  Social involvement | Demographic  (age,  sex,  Marital status  Social structure :  Education  (Family structure(  Two parent, single parent, other)  [Occupation]  School/work involvement (proportion), | -------- | 134,072  Canadians | 21.  Bhatti, T. et al. 2007, canada |
| NO | dental care utilization in the last month  concentration index for dental care utilization | ------ | ------ | Wealth index  Insurance coverage  Geographical area  (urban/rural) | Demographic variables  (Sex  Age  Marital status )  Social structure:  (Education  Beliefs ( | -------- | 37860  Cross sectional survey | 22. Rezaei S. et al. 2019, IRAN |
| NO | ---- | ----- | Evaluated or Normative need:  functional dentition  (number of natural teeth)  eating comfortably  socialising without embarrassment | ---- | Demographic :  (Age ,  Sex  Social structure:  Education,  Occupational class ,  Subjective social status(Highest rank, second highest rank, second lowest rank, lowest rank)  Beliefs | Contextual predisposing : demo and social  (socio economic inequalities  1.Relative Index of Inequality (RII)  2.Slope Index of Inequality (SII) ) | 31 country  cross-sectional study | 23. Guarnizo-Herreño CC. et al. 2013, UK |
| Relatively | Reason for last visit  Usual reason for dental visit  Time since last visit  12 month,  1-2 y,  2-5y,  5-10y,  >10y  Usual frequency of dental visiting  Has usual source of dental care | ----- | Self-rated dental health  Estimated value of lost productivity due to dental problems  ------- | Household Income  Cardholder status  Insurance status  Remoteness location  (region of country or state or city) | Demographic :  Age  Gender    Social structure: | ------ | 6284  Cross sectionsl survey  National Dental Telephone Interview Survey (NDTIS) | 24.  Harford J, et al. 2012, Australia |
| NO | Regularity of dental check-ups  Less than 1 y  ,  1y  Less than 2y  ,  2y  Treatment type  waiting time (month) | ----- | ---- | Net monthly income per persons in the same household | Demographic  (Age,  Sex  social structure:    Education ,  Working status ,  Retirement status  Beliefs : | ------ | 210  Cross sectional study | 25.  Tuominen R. et al. 2012, Finland |
| NO | Use of dental check-up in the last 12 months | ----- | ------- | Socioeconomic status  Type of health insurance  Place of residence(east/west)  Urban residence  (urban/rural) | Demographic:  (Sex,  Age groups,  Living together,  Social structure:  (Migration status, | ------ | 22,050  cross-sectional survey | 26.  Erdsiek F. et al. 2017, Germany |
| Relatively | Visiting a dentist ?  Choice of dentist ?  Number of visits  in the study year  Sufficient public services  Sufficient private services  Recall  Visit time (required for a visit to the dentist, including travel, waiting and treatment time )  Dentist density | ------- | Pain    All natural teeth  number of missing teeth | Income | Demographic  (Age , gender )  Social structure:  (Education ,  [occupation]  Unemployment, Student,  Other occupation,  Beliefs (Fear, | ------ | 2010  Cohort study | 27.  Nguyen L. et al. 2006, Finland |
| Relatively | Dental visits  during the past 12 months | ------ | Toothache  Missing teeth | Economic activity | Demographic  (Gender  , Age ,  Social structure:  Education ,  Ethnicity |  | in Estonia  n=13,750)  in Lithuania  (n = 9034)  cross-sectional survey | 28. Leinsalu M et al. 2018, Estonia and Lithuania |
| Relatively | Dental visit annually  Time to get to dental office  <= 30 min  >30 min  Patient satisfaction (DVSS)  Dental Visit Satisfaction Scale | Alcohol intake  Preventive behavior  Exercise  Smoker  Social network | Evaluated and perceived need :  Bleeding gums  Satisfied with own teeth  Perceived condition of teeth  No. of teeth present | Yearly income (DKK)  Costs of importance    Private dental insurance | Demographic :  Age ,  Sex  Social structure:  Education  Beliefs :  Corah’s Dental Anxiety Scale (CDAS) | --------- | 800  Cross sectional study | 29.  Scheutz F. et al. 2001,  USA |
| Relatively | Dental service use in the past 12 month | -------- | Perceived need :  Problem with teeth or gums  Self-rated oral health | Dental health insurance | Demographic: (Age ,  Gender,  Marital status    Social structure:  Education ,  Region by  ethnic density (florida, Hawaii, texas, New York, california) | ------ | 2128  Cross sectionsl survey | 30. Jang Y. et al. 2019, USA |
| Relatively | Annual Dental Visit last year | ------- | Self-reported health status | Income  Mean number of dentists per 100,000 | Demographic:  Age,  Gender  Social structure:  Education Attainment, ,Race/Ethnicity | ------- | 477,665  Cross sectional Survey | 31. Chou C.F. et al. 2019, USA |
| Relatively | Past year dental health utilization? | Smoking status  Vegetable servings/day  Fruit servings/day | self-rated health status | Income  Insurance  Place of sick care  Frequency of Social support | Demographic:  (Age,  Gender ,  Marital status,  Social structure:  Education  Employment status  Beliefs: ----- | ------- | 1,444  Cross sectionsl survey | 32.  Stapleton S. et al. 2016, USA |
| NO | ----- | ------- | Oral impacts on daily life  (frequency of impacts of oral conditions on daily life)  1.Difficulties in eating food,  2.Difficulties in chewing/biting foods,  3.Experiencing toothache/painful gums/sore spots,  4.Feeling tense,  5.Feeling embarrassed,  6.Avoiding conversation  7.Reducing participation in social activities. | Subjective social status(SSS) | Demographic: Age ,  Sex  ,Marital status  Social structure:  Education,  Occupational class  Beliefs: | ------- | 21731  cross-sectional survey | 33. Guarnizo-Herreno CC. 2014, UK |
| NO | ----- | ------ | Evaluated need:  DMF index  Inequality indec:  Concentration index  If all the people in various socioeconomic groups have the same health status, the concentration index will be zero and the concentration curve will be tangent to the line of equality. | Family:  SES quintile  district of residenc  (1,  2,  3(high SES)  Insurance status | Demographic:  (Age ,  Marital status )  Social structure:  Education  Beliefs: | ------- | 2000  cross-sectional study | 34.  Moradi GH. et al. 2017, IRAN |
| Relatively | Have you ever had a dental visit? | ------ | perceived Need  Perceived dental treatment  evaluated Need  Decayed teeth | Family monthly income (R$) | Demographic:  Age, Sex  social structure:  Skin color  (was employed to assess ethnicity )  Years of schooling | Contextual  predisposining demographic  HDI-Longevity (2000)  Low  Moderate  High  Predisposing contextual demographic was evaluated based on the life expectancy of the city according to the component Longevity of the Human Development Index (HDI)  Predisposing social  Predisposing social included the component Education of the HDI and the Gini Index.  HDI  Enabling health policies  OHT/FHS  Low  Moderate  High  Oral Health Team (OHT)  Family Health Strategy (FHS)  The contextual variable of the municipality was represented by the estimate of the population coverage of the Oral Health Team (OHT) of the Family Health Strategy (FHS) for 2013,  Enabling financing  HDI-Income (2000) | 7,265  cross-sectional study | 35.  Rebelo Vieira J.M. et al. 2018, Brazil |
| Relatively | Time Since Last Dental Visit?  1 year or less %(N)  More than 1 year ago % (N)  Unmet dental care need  Reasons for unmet dental care need | ------ | General health status | Income status  by poverty income ratio (PIR)  Health insurance | sociodemographic :  Age ,  Ethnicity/race ,  Marital status  Social structure:  Education,  Ethnicity/race | ----- | 3,790  weighted  Cross  sectional surveys | 36.  Gupta A. et al. 2019, US |
| NO | dental service utilization in the year preceding the ED visit  [ the past year] | ------ | ------- | Median household Income by zip code of residence  Payer type  (Commercial  Insurance  Medicaid  Medicare  Self-pay  Other)  Residing in a dental health professional shortage area  Dental coverage at time of ED visit | Demographic:  Age,  Gender  Social structure:  Beliefs: | ------- | 1,843,273  Cross sectional survey | 37. Ranade A. et al. 2018, US |
| NO | When do you usually visit a dentist?  1 = “Regularly for check-up,”  2 = “Only when having pain or some problem”  3 = “Never.” | ----- | ------ | ------ | Demographic:  Age  Gender  Social structure:  Education  Beliefs:  How afraid are you of visiting a dentist?  1 = Not at all  2 = Somewhat  3 = Very much  dental fear?  stable no-fear,  stable high fear,  decreased fear  increased fear | ------ | 3631  Cohort study | 38.  Liinavuori A. et al. 2019, Finland |
| Relatively | When was your last dental appointment?  1. In the last 12 months;  2. From one year to less than two years;    3. From two years to less than three years;  4. Three years or more;  5. Never | ------- | Need perceived:  Perceived dental health  Eating difficulties due to oral problems  Need evaluated  Missing teeth  (No  One or more  All) | Income  Health insurance  Registered in FHT  (Family Health Teams )  (in  locations covered by the FHT in primary care, increase liklihood of dental utilization) | Demographic:  Age, Sex,  Social structure:  Years of schooling,  Social network  Race/skin colour ,  Beliefs: | Contextual Predisposing demographic  HDI: Human Development Index,  HDI—life expectancy  HDI—education  data on income, education and life expectancy from the Human Development Index obtained from the Atlas of Human Development in Brazil,  Contextual Enabling  HDI—income    1.Per capita expenditure in primary care  2.Per capita expenditure in oral care  3.programs  Family oral health teams coverage  Contextual Perceived need  Oral Impacts on Daily Performances,  Dental pain  (OIDP questionnaire)  Contextual Evaluated need  DFMT index  Need for denture  (OIDP questionnaire)  Percentage of extremely poor  Percentage of vulnerable to poverty  Contextual (city-level)  1.Predisposing (greater scores indicate better conditions)  2.Enabling (greater scores indicate better conditions)  3.Perceived need (greater scores indicate worse perceived oral health)  4.Evaluated need (greater scores indicate worse oral health) | 27,017  cross-sectional survey | 39.  Herkrath F.J. et al. 2018, Brazil |
| Relatively | Utilisation of dental services  (Never been to the dentist  ≥ 3 years since last dental visit  2 to < 3 years since last dental visit  1 to < 2 years since last dental visit  < 1 year since last dental visit | ------- | Perceived Need-related variables  Self-rated oral health  Eating difficulties due to oral problems  Self-reported tooth loss  (mean, 95%CI)  (ranging from 0 = no tooth lost to 32 = lost all teeth) | Personal income  Number of residents in the household  Number of rooms in the household  Durable goods and items  Health insurance  Duration of health insurance  Registration in the primary care (increase access to dental care) | Demographic :  Age ,  Sex  Social structure:  Education ,  Lives with spouse or partner  Number of close relatives    Number of close friends  Participation in sports or artistic group activities  Participation in community meetings and associations  Participation in voluntary services (unpaid)  Participation in religious meetings  Beliefs: | ------ | 60,202  Cross sectional survey | 40.  Herkrath F.J. et al. 2020, Brazil  R |
| Relatively | How often do you visit a dentist for dental care?  “never” = 0, “rarely” = 1, “less than once every two years” = 2, “at least once every two years” = 3, “at least once a year” = 4, or “twice a year” = 5.  Regular physical exam? | ---- | Self-rated health  Tooth loss  Gum bleeding | Income  Health insurance coverage | Demographic:  Gender , Age  Marital status  social structure:  Education  Beliefs:  Care about healthy food? |  | Cross sectional survey | 41.  Qu X. et al. 2020, China |
| NO | History of receiving dental care?  “How often do you usually visit the dentist and when was your last dental visit?”  (23)  Solutions for improving dental care utilization in pregnancy  1.Knowledge  2.Financial support  3.Establishing supportive policies | ----- | Experience of dental caries and periodontal diseases before and during the pregnancy?  “How would you describe your dental and oral status before and during pregnancy?” | ----- | Demographic :    How old are you?  Social structure:  What is your educational level?  Beliefs:  Beliefs about dental care during pregnancy ?  “What is your opinion about visiting the dentist or dental procedures during pregnancy?”  Reasons if no visits were reported during pregnancy and what solutions could facilitate receiving dental services?  “What causes you to neglect dental checkup during pregnancy and what factors can facilitate it? | ----- | Qualitative study  triangulation approac | 42.  Bahramian H. et al. 2018, IRAN |
| Relatively | n the past year, have you seen a dentist?  Concentration index for inequality assessment | ----- | Perceived need :  Self-reported health status | [Income,]  Pce:  The household final consumption expenditure per capita (Pce)  Types of medical insurance  Area :  (urban/rural)  Western/eastern/  Northeast/cental | Demographic:  Age,  Gender,  Marital status,  Social structure:  Education  Beliefs: | ------ | 17 648 +  15 450 =  17 648 individuals aged 45 years and older in 2013 and 15 450 individuals in 2015 who participated in the China Health and Retirement Longitudinal Study (CHARLS.)  National cross sectional Survey | 43.  Li C. et al. 2018, China |
| Relatively | Have you ever visited a dentist?  Yes / No | Personal health practice:  Tooth cleaning frequency  ≤Once/day  ≥Twice/day    Tooth cleaning method  Toothbrush  Other  Fluoridated toothpaste use  Yes  No  Smoking  Yes  No  Tobacco chewing Yes  No  Alcohol consumptiona  Yes  No | Evaluated need :  Periodontal disease None-mild  Moderate-severe  DFT  (decay and fill teeth)  No  Yes  Missing teeth  No missing tooth  ≥1 missing teeth | Per-capita income Low-middle  High    Material circumstances  Poor  Good | Demographic:  Age group  35-44  45-54  Sex  Male  Female  Social structure:  Education level  Secondary/less  Post-secondary | ----- | 873  Cross sectional study | 44.  Bhat M. et al. 2017 India |
| Relatively | Use of dental health services (Latent variable) Utilization  Reason for attending dentist  Do not attend/attend seldom  When problem (pain, lost fillings)  Having routine recall/ check-up  Attendance to dental health care services  Only when having problems  Longer intervals than 2 y  Every second year  Every year | Toothbrushing  Less than once a day  Once a day  Twice a day  Frequency of sugary soda drink intake (Observed variable)  Seldom  A few times every week  Several times a week to daily | Self-perceived treatment need  Would not need treatment  Don't know  Evaluated need:  Clinically evaluated outcome  Number of decayed teeth (Observed variable) | Incom  Sense of Coherence (SOC)  Urbanization  (the number of inhabitants and availability of dentists as a ratio of  inhabitants per dentist)  Refrained from dental check-up due to lack of money  Difficult accessing routine care | Demographic  Age  20-79  Gender  Male / female  Social structure:  education  beliefs:  Dental Anxiety Scale | ----- | 1840  cross-sectional study | 45.  Jonsson B. et al, 2020  Norway |
| NO | Last dental check-up  Less than 2 years ago  More than 2 years ago | ----- | ---- | Income per consumption unit  1st quartile > 2,605  2nd quartile >1,733 & <= 2,605  3rd quartile > 1,115 & <= 1,733  4th quartile <= 1,115  Health insurance  Social security + top-up cover  CMU + CMU-C  CMU or social security alone  Don’t know/No health coverage | Demographic:  Gender  Men  Women  Age  18–29 y/o  30–44 y/o  45–59 y/o  60 y/o and older  Social structure:  Origin  French  French with an immigrant background  Foreigners  Education  level Bachelor degree  High school diploma  Lower secondary education  Socio-professional group  Manager, intellectual profession  Intermediate profession  Craftsman, trader    Employee  Worker  Has never worked | ------- | 2997  A cross-sectional analysis | 46.  Trohel G. et al. 2016 USA |
| NO | ‘Have you visited a dental clinic in the past year?’ | ------ | ----- | Economic activity status  No / yes  Income  Low  Middle-low  Middle  Middle-high  High  Regional level  1  2  3  4  5  deprivation index | Demographic  Age (y)  19-29  30-39  40-49  50-59  60-69  >70  Gender  Men  Women  Cohabitation status with spouse  Yes / No  Social structure:  Education:  No formal  Elementry  Middle-high  University or higher  Number of household member | ------ | 220 258  Cross sectional survey | 47.  Cheol-S. K. et al, 2015 south korea |
| Relatively | Dental visits  t in the previous 12 months  Yes / No | Regular physical exam  Yes / No  How concerned about healthy food  Not at all  Moderately  Extremely  Smoker  Yes / No  Drinker  Yes / No  Exercise regularly  Yes / No | Health rated status  as  fair / poor  (N, %)  Number of limitations resulting from chronic conditions  (N)  Number of ADL and IADL assistances received  ADL,  activity of daily living; IADL, instrumental activity of daily living  (range: 0-11) | Monthly income  Social medical insurance coverage  Yes / No  Collective medical insurance coverage  Yes / No  Ability to pay out-of-pocket expenses yes / No | Sociodemographics Age (range: 60-103 years)  Female  N, %  Living alone  Yes / No    Social structure:  Middle school or above  Yes / No  Beliefs:  Overall life satisfaction  (range: 3-15) | ----- | 1044  Cross sectional study | 48.  Bei W. 2007 CHINA |
| NO | dental  visit within the past 12 months | ----- | ----- | Household income  Low  Medium  High  Insurance status  Non-insured  Public insurance    Commercial insurance | Demographic :  Age  18–24  25–34  35–44  45 +  Gender  Men/ women  Social structure:  Level of education  Low  Medium  High | ----- | 1086  cross-sectional study  phone interviews. | 49. Bayat F.  Et al. 2006 IRAN |
| Relatively | having a past-year dental visit?  Yes / No | Prevent tooth decay  Other  Brushing and Flossing  Tooth brushing frequency  Twice or more  Once a day  Use of dental floss  No  Yes | perceived need:  self reported  Need OH treatment  Yes / NO  OH problems  Yes / NO  Oral Health condition [status]  Good  poor | Employment status  Yes / NO  Medical insurance  Yes / NO  Place Medical Care  Community Clinic  Other  Social support  Yes / NO  Acculturation  Strong  Weak  Acculturation was measured with a 20-item modified Bicultural Acculturation Scale. | Demographic:  age  18–49  50 and older    Sex  Female  Male  Social structure:  Country of origin Place  Other  Mexico  Formal education  No  Yes  Relationship status  No  Yes  Religious beliefs  No  Yes | ------- | 278  Cross-sectional study | 50. Serna CA. et al  2020,  USA |
| NO | Oral health service utilization in past 12 mo  Yes  No  Reasons  Consultation and check‐up  Prevention  Treatment  Unknown | ----- | ------- | Location  Rural  Urban  Annual household income per capita  1st tertile (low)  2nd tertile (medium)  3rd tertile (high)  Medical insurance coverage  NCMS  URBMI  UEBMI  GMI  No basic medical insurance  Private medical insurance  No  Yes  Abbreviations: GMI, government medical insurance; NCMS, New Cooperative Medical Scheme; UEBMI, Urban Employee Basic Medical Insurance; URBMI, Urban Resident Basic Medical Insurance. | Demographic:  Sex  Male  Female  Social structure:  Educational attainment level  Low  Moderate  High | ------ | 7206  Cross sectional survey  National Oral Health Survey | 51. Xu M. et al. 2019 CHINA |
| Relatively | Time interval since the most previous dental visit 12 months  <6 months  6–12 months  >12 months | ------- | Evaluated need:  Number of teeth lost  0–2  3–9  10 +  Data missing for 6 cases | Income  Above average  Average  Below  Average  City of residence  ( Vilnius Kaunas Klaipėda Šiauliai) | Demographic :  Gender  Women  Men  Marital status  Cohabiting  Single  Social structure :  Education  University  Less than university | ------ | 862  Cross sectional study | 52. Sakalauskienė Z. et al 2009 Lithuania |
